# Periodontal status following orthodontic mini‐screw insertion: A prospective clinical split‐mouth study

**DOI:** 10.1002/cre2.757

**Published:** 2023-06-20

**Authors:** Negar Moeini, Hamoun Sabri, Pablo Galindo‐Fernandez, Hoorieh Mirmohamadsadeghi, Nasrin Keshavarz Valian

**Affiliations:** ^1^ Department of Periodontics Shahid Beheshti University of Medical Sciences School of Dentistry Tehran Tehran Iran; ^2^ Department of Periodontics and Oral Medicine University of Michigan School of Dentistry Ann Arbor Michigan USA; ^3^ Center for Clinical Research and Evidence Synthesis in Oral Tissue Regeneration (CRITERION) Ann Arbor Michigan USA; ^4^ Oral Surgery and Implant Dentistry Department, School of Dentistry University of Granada Granada Andalucía Spain; ^5^ Department of Orthodontics Shahid Beheshti University of Medical Sciences School of Dentistry Tehran Tehran Iran

**Keywords:** mini‐implants, mini‐screw, orthodontic anchorage, periodontal health

## Abstract

**Background:**

Anchorage control is one of the most important determinants of orthodontic treatments. Mini‐screws are used to achieve the desired anchorage. Despite all their advantages, there is a possibility that treatment will not be successful due to conditions related to their interaction with the periodontal tissue.

**Objective:**

To evaluate the status of the periodontal tissue at the sites adjacent to the orthodontic mini‐implants.

**Methods:**

A total of 34 teeth (17 case and 17 control) in 17 orthodontic patients requiring a mini‐screw in the buccal area to proceed with their treatment were included in the study. Oral health instruction was provided to the patients prior to the intervention. In addition, scaling and root planing of the root surface were done using manual instruments and ultrasonic instruments if needed. For tooth anchorage, a mini‐screw with Elastic Chain or Coil Spring was used. The following periodontal indices were examined in the mini‐screw receiving tooth and the contralateral tooth: plaque index, pocket probing depth, attached gingiva level (AG), and gingival index. Measurements were made before the placement of the mini‐screws and 1, 2, and 3 months following that.

**Results:**

The results revealed a significant difference only in the amount of AG between the tooth with mini‐screw and the control tooth (*p* = 0.028); for other periodontal indices, there were no significant differences between the two groups.

**Conclusion:**

This study showed that periodontal indices in adjacent teeth of the mini‐screws do not change significantly compared to other teeth and mini‐screws can be used as a suitable anchorage without posing a threat to the periodontal health. Using mini‐screws is a safe intervention for orthodontic treatments.

## 1 INTRODUCTION

Anchorage control is one of the most crucial key factors in orthodontics dictating the desired outcome of the treatment (Rodriguez et al., 2014). Traditionally, adjacent teeth and intra‐ and extraoral devices have been used routinely for orthodontics anchorage; nevertheless, the use of these devices is mostly dependent on patient compliance and therefore, could be unideal most of the time (Molina‐Solana et al., 2013). In modern orthodontics, mini‐screw implants are being used to achieve the desired anchorage in the treatments (Leung et al., 2008). Various studies have evaluated the outcomes of the treatment using these devices and reported the ideal outcomes of using so in treatment modalities such as distalization, intrusion, extrusion, protraction, midline correction, and occlusal plan adjustment (Abdulnabi et al., 2017; Yanagita et al., 2011). The most important benefit of using mini‐screws comprises easy application, low cost, biocompatibility, less‐invasive nature of placement and removal, and independency from the patients' compliance (Alharbi et al., 2018; Cousley & Sandler, 2015; Prabhu & Cousley, 2006). In a systematic review by Alharabi et al., (2018) the failure rate of the treatment using mini‐screws was reported between 12.5% and 14.3%.

On the other hand, despite the mentioned benefits, several studies have reported the risk indicators of failure of these mini‐implants (Baek et al., 2008; Casaña‐Ruiz et al., 2020; Severo & Barbosa, 2015). These include periodontal inflammation, gingivitis, and proximity of the inserted mini‐screws to the root surfaces. Also, mini‐implants placed in the maxillary arch as well as placement in the areas with a lack of keratinized tissue have also been shown to be more prone to failure (Casaña‐Ruiz et al., 2020; Moon et al., 2008; Viwattanatipa et al., 2009). Moreover, poor oral hygiene and smoking are also reported among the failure risk indicators (Kravitz & Kusnoto, 2007). As these mini‐screws can be identified as a foreign body, they could contribute to plaque accumulation and therefore, inflammation of the surrounding tissue which increases the risk of the failure in orthodontic treatment (Bae et al., 2002). Furthermore, Chen et al. (2008) reported damage to the periodontal ligament (PDL) and resultant root resorption following the placement of the mini‐screws. Overall, the current evidence agrees on possible risks for periodontal health while using mini‐implants as anchorage units via possible localized gingival inflammation and risk of infection if poor oral hygiene is performed. Additionally, the potential retention of food debris may induce bacterial proliferation as well as plaque formation which threatens the patients' periodontal health also the orthodontic treatment success (Mohamed et al., 2023). A recent study by Liu et al. (2023) indicated significantly increased bleeding scores and plaque accumulation. Subsequently, these make it crucial to occupy a meticulous and strict oral hygiene strategy throughout the mini‐implant anchorage phase in orthodontic treatment.

Gingival inflammation around the tooth is known as gingivitis, which is a periodontal disease and it has been shown that fixed orthodontic appliances can invade the periodontal tissue and lead to difficulties in plaque removal and proper oral hygiene thereby, affecting periodontal health negatively (Imano et al., 2020; Ong & Wang, 2002). The resultant inflammation could proceed to aggravate the situation and cause periodontitis. This in fact can turn into a double‐edged sword, causing further damage to patients' periodontal health as well as compromising the anchorage outcomes. Numerous studies evaluated the effect of fixed orthodontic appliances on periodontal health status (Alfuriji et al., 2014; Atassi & Awartani, 2010; Karkhanechi et al., 2013; Miethke & Vogt, 2005; Sadowsky & BeGole, 1981); yet, the literature evaluating the same variables in patients treated with mini‐screws is scarce (Bayani et al., 2015). Therefore, in this study, we aimed to evaluate the periodontal health status adjacent to the implanted orthodontic mini‐screws as orthodontic anchorage in a split‐mouth fashion, to reveal any possible negative impact on the periodontal tissue.

## 2 MATERIALS AND METHODS

### 2.1 Study design and protocol

This study was conceptualized within a prospective split‐mouth case‐control study. Before the initiation of the study, the protocol was approved by the ethical committee of the Shahid Beheshti University of Medical Sciences Research Institute with the approval code of IR. SBMU. DRC. REC.1398.137. Also, the present research was conducted in full accordance with ethical principles, including the Declaration of Helsinki of 1965, as revised in 2013. Likewise, the present manuscript follows the STROBE statement for improving the quality of reports of observational studies (http://www.strobe-statement.org/). The corresponding checklist is provided in Supporting Information: Appendix A.

All the patients requiring orthodontic treatment using implanted mini‐screws for anchorage, who were presented to the Department of graduate orthodontics Shahid Beheshti University dental school from March 2020 to March 2021 were screened for the inclusion criteria as follows:

1‐ Healthy adult patients, presenting mild to moderate Class II malocclusion and minimal crowding (<3 mm) in the maxillary arch.
2‐ Intact maxillary arch dentition (with or without third molars).
3‐ Indication for placement of unilateral mini‐screws for orthodontic anchorage to obtain molar distalization.
4‐ Patients without active periodontal disease (Chapple et al., 2018).

On the contrary, the exclusion criteria were:

1‐ Presence of any systemic disease or conditions, which might affect the bone remodeling and healing process negatively.
2‐ Patients receiving any medications which can affect bone remodeling negatively.
3‐ Indication of antibiotic prophylaxis.
4‐ History of radiotherapy.
5‐ Presence of infection on the intervention site.
6‐ Presence of any type of prosthetic device at the intervention site.
7‐ Invasion of the mini‐screws to the PDL.
8‐ Presence of the symptoms of Gingivitis or Periodontitis.

### 2.2 Subjects recruitment

Based on the defined inclusion and exclusion criteria, the patients requiring the mini‐screws implantation on one buccal side of their first molars were selected for the study. Moreover, written consent was obtained from the patients/parents following giving the instructions and information regarding the present study.

### 2.3 Clinical process

Before the intervention, oral hygiene instructions (OHI) were given to the subjects (tooth brushing with the Modified Bass technique and a medium bristled toothbrush and the proper technique of flossing the mini‐screws). Subsequently, the patients underwent scaling and root planing using both hand and ultrasonic instruments. For the tooth movements, a mini‐screw was used with either an elastic chain or coil spring.

### 2.4 Mini‐screw insertion and force delivery

One bracket‐type mini‐screw (Absoanchor, Dentos) with a diameter of 1.1–1.4 mm and a length of 7 mm was placed for each patient to facilitate retrusion movement of the anchor unit. The proper location of inserting the mini‐screws was evaluated according to peri‐apical radiographs of the area. Following the injection of local anesthesia, the recipient site was prepared by a pilot drill with 400–500 rpm. Next, using a hand‐piece screw‐driver, the mini‐implants were placed. After the insertion, another peri‐apical radiograph was obtained to confirm the correct position. The complete OHI was given to the patient. A 2‐week interval of healing was given to the patients and subsequently, the loading phase was started. Depending on the required movement, the amount of force was adjusted with a gauge and it was confirmed to not exceed 50 g. The tipping movement was planned to achieve by engaging the wire within the slot. The patients were planned to be revisited in 1‐, 2‐ and 3‐month follow‐ups.

### 2.5 Intraexaminer calibration

In the present study, all the measurements were done by one investigator (N. M.). To evaluate the interexaminer calibration, the measurements of pocket depths in 24 teeth in 8 patients were measured at two different time points using William's periodontal probe and Interclass Correlation Coefficient (ICC) analysis was performed to evaluate the interexaminer reliability. The value of ICC was 0.92, 1, and 0.8 for mesial, distal, and mid‐buccal, respectively. Thus, a high similarity between the values was observed.

### 2.6 Evaluation of the periodontal indices

Various periodontal tissue health indices were evaluated before the placement of the mini‐screws and at the 1‐, 2‐, and 3‐month follow‐up visits.

- **Attached Gingiva (AG)**
  The width of the AG, from the most coronal AG margin to the mucco‐gingival junction, was measured using a periodontal probe in the buccal aspect.
- **Gingival Index (GI)**

It was measured based on the Löe and Silness (1963) method to evaluate the presence of gingival inflammation. This was evaluated in four gingival units of the tooth (disto‐facial, mid‐facial, mesio‐facial, and lingual) using a periodontal probe and scored as follows:

1. Normal gingiva; without discoloration and bleeding on probing (BOP) and inflammation.
2. Mild inflammation; slight discoloration hence, negative BOP.
3. Moderate inflammation; erythematous gingiva, swelling, edema, and positive BOP.
4. Severe inflammation; erythematous gingiva, severe swelling, edema, and positive BOP.

By dividing the sum of all four areas of each tooth by four, the GI of each tooth was measured.

- **Plaque Index (PI)**

The PI was measured using a periodontal probe in four areas of the tooth as follows (Löe & Silness, 1963):

1. Absence of any plaque.
2. A thin layer of plaque near the free gingival margin and detectable with an explorer.
3. Moderate plaque accumulation near the free gingival margin. Absence of plaque in the interdental areas.
4. Considerable amounts of plaque accumulation in the interdental areas and free gingival margin.

- **Pocket Depths (PD)**

Measured using William's probe. The probe was kept parallel to the long axis of the root on the interproximal, buccal, and lingual areas.

### 2.7 Statistical analysis

In the present study, a comprehensive statistical analysis was conducted to investigate the data obtained from the research participants. First, the assumption of normal distribution was assessed for the variables of interest using the Kolmogorov–Smirnov test. Levene's test was used to evaluate the homogeneity of variances between groups. The paired *t*‐test was employed to examine within‐group differences over time for each variable separately. Additionally, a two‐way analysis of variance was conducted to assess the interaction effect between the independent variables (test or control) and the dependent variables (outcome measures) across the two time points. Posthoc tests were used for pairwise comparisons when a significant main effect or interaction effect was observed. The baseline characteristics of the groups were examined using independent samples *t*‐tests. Any significant differences at baseline would be reported and taken into consideration in the interpretation of the study results.

All formal statistical analyses were performed by one author (H. S.). The SPSS version 20 software (SPSS Inc.) was used for the data analyses. Variables with a *p* < 0.05 were considered statistically significant.

## 3 RESULTS

This observational study was conducted on 17 patients (11 Females and 6 males, mean age of 24.52 ± 6.11 years) and 34 teeth. A total of 17 first molars for the anchoring group and 17 contra‐side first molars were included as the control group to be evaluated. All of the included first molars were located in the maxillary arch. The descriptive data of the subjects are presented in Table 1. The patients completed the orthodontic treatment phase without any complications. The mean value and the frequency of the measured variables for the case and control groups are described in Table 2.

**Table 1:** Descriptive characteristics of the subjects.

Parameter	Value
Subjects	17 (11 male)
Age	24.52 ± 6.11 years
Anchoring teeth	17 (first molar)
Control teeth	17 (contra‐site first molars)
Arch	Maxilla (17)

**Table 2:** Periodontal indices (mean ± SD) for the case (mini‐screw) and control groups on the baseline and at the end of 3rd month follow‐up.

Periodontal health variable	Group	*N*	T0		T3		*p* Value	
			Mean	SD	Mean	SD	Intragroup	Intergroup
Plaque index	Case	17	0.7426^a^	0.14968	0.738	0.16	0.742	0.485
	Control	17	0.7794^a^	0.14843	0.72	0.14	0.874	
Gingival index	Case	17	1.2132^a^	0.25297	1.24	0.27	0.774	0.680
	Control	17	1.1838^a^	0.19822	1.21	0.22	0.699	
Attached gingiva	Case	17	3.89^b^	0.37	3.29	0.85	0.882	0.028*
	Control	17	4.11^b^	0.65	3.62	1.10	0.741	
Mesial PD	Case	17	3.3382^a^	0.69564	3.52	0.72	0.953	1.000
	Control	17	3.3382^a^	0.70678	3.41	0.69	0.256	
Distal PD	Case	17	2.8529^a^	0.64987	2.81	0.66	0.588	0.711
	Control	17	2.9265^a^	0.59794	2.89	0.58	0.746	
Lingual PD	Case	17	1.8235^a^	0.64205	1.82	0.58	0.973	1.000
	Control	17	1.8235^a^	0.58473	1.94	0.48	0.142	

Abbreviations: PD, pocket depth; SD, standard deviation.

^a^ No statistically significant difference at baseline between the two groups (*p* > 0.05) (Independent samples *t*‐test).

^b^ Statistically significant difference between the two groups (*p* < 0.05) (Independent samples *t*‐test).

* indicates a significant *p* value (<0.05).

As the result of the independent samples *t*‐test indicated, at the baseline, there were no significant differences in any of the variables (PI, GI, and PD) except AG (*p* = 0.04).

**Figure 1:**
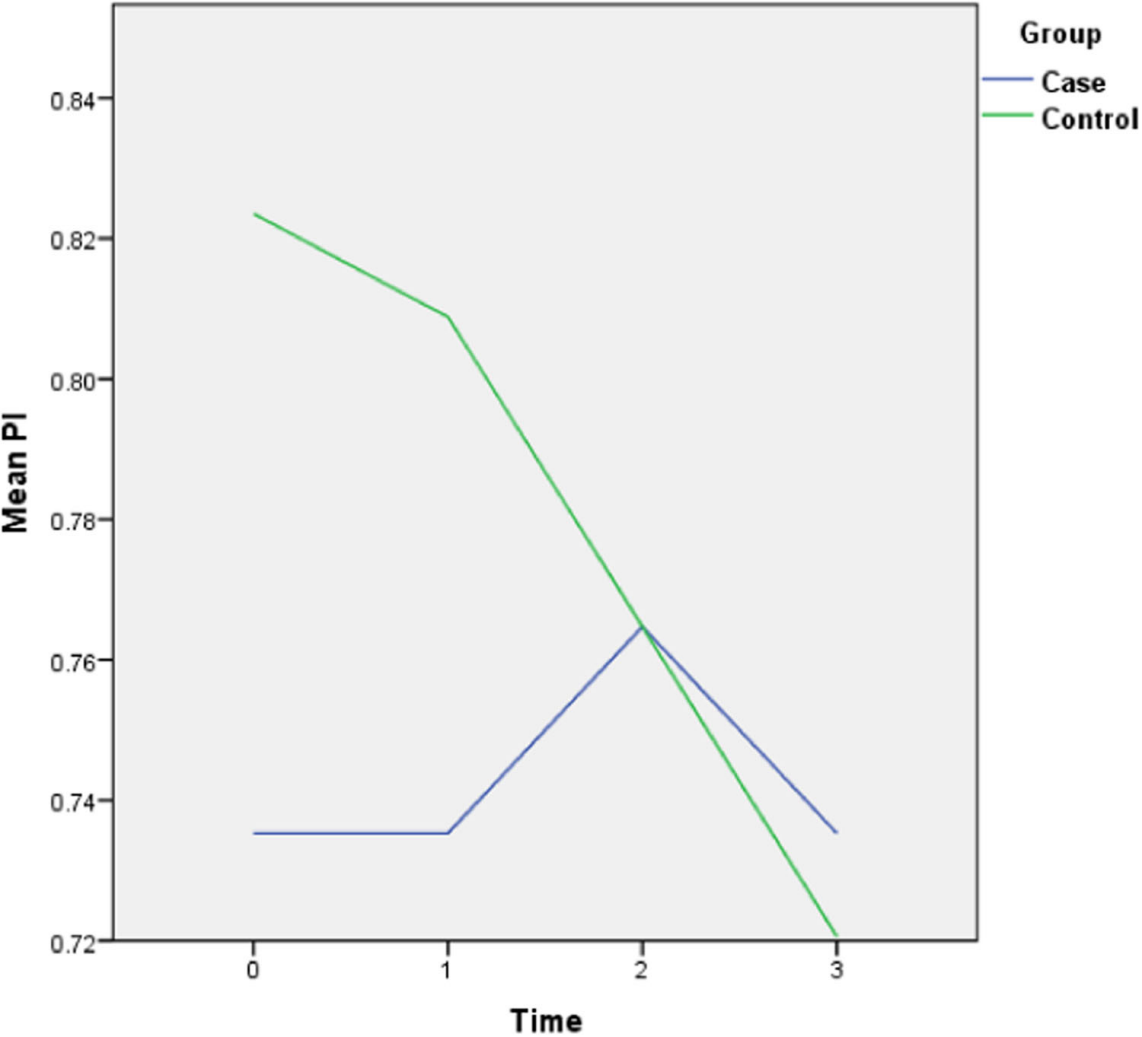
Plaque index (PI) alterations throughout the follow‐up period. (0 = Before placement of the mini‐screws, 1, 2, and 3 months following that.)

**Figure 2:**
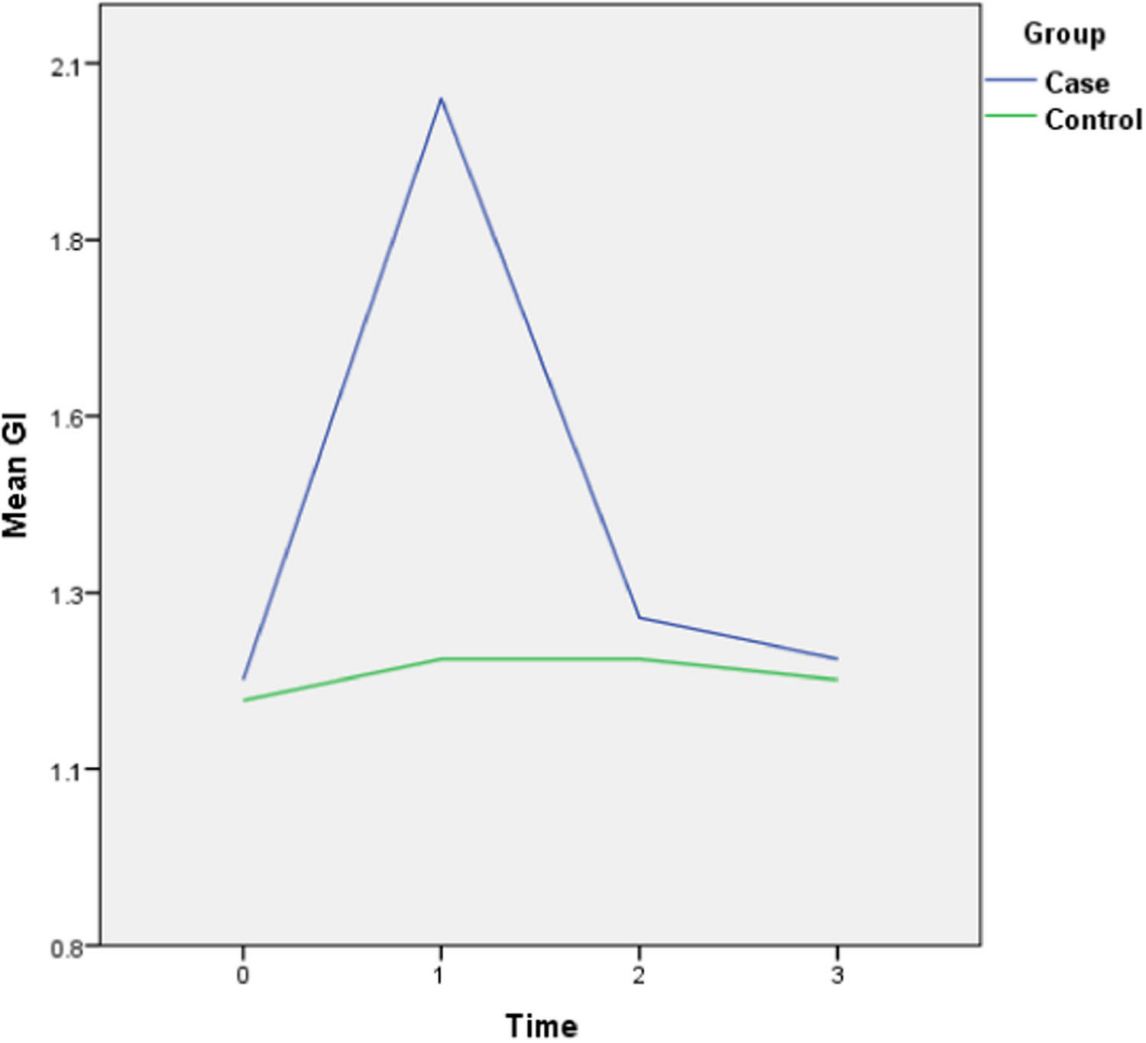
Gingival index (GI) alterations throughout the follow‐up period. (0 = Before placement of the mini‐screws, 1, 2, and 3 months following that.)

**Figure 3:**
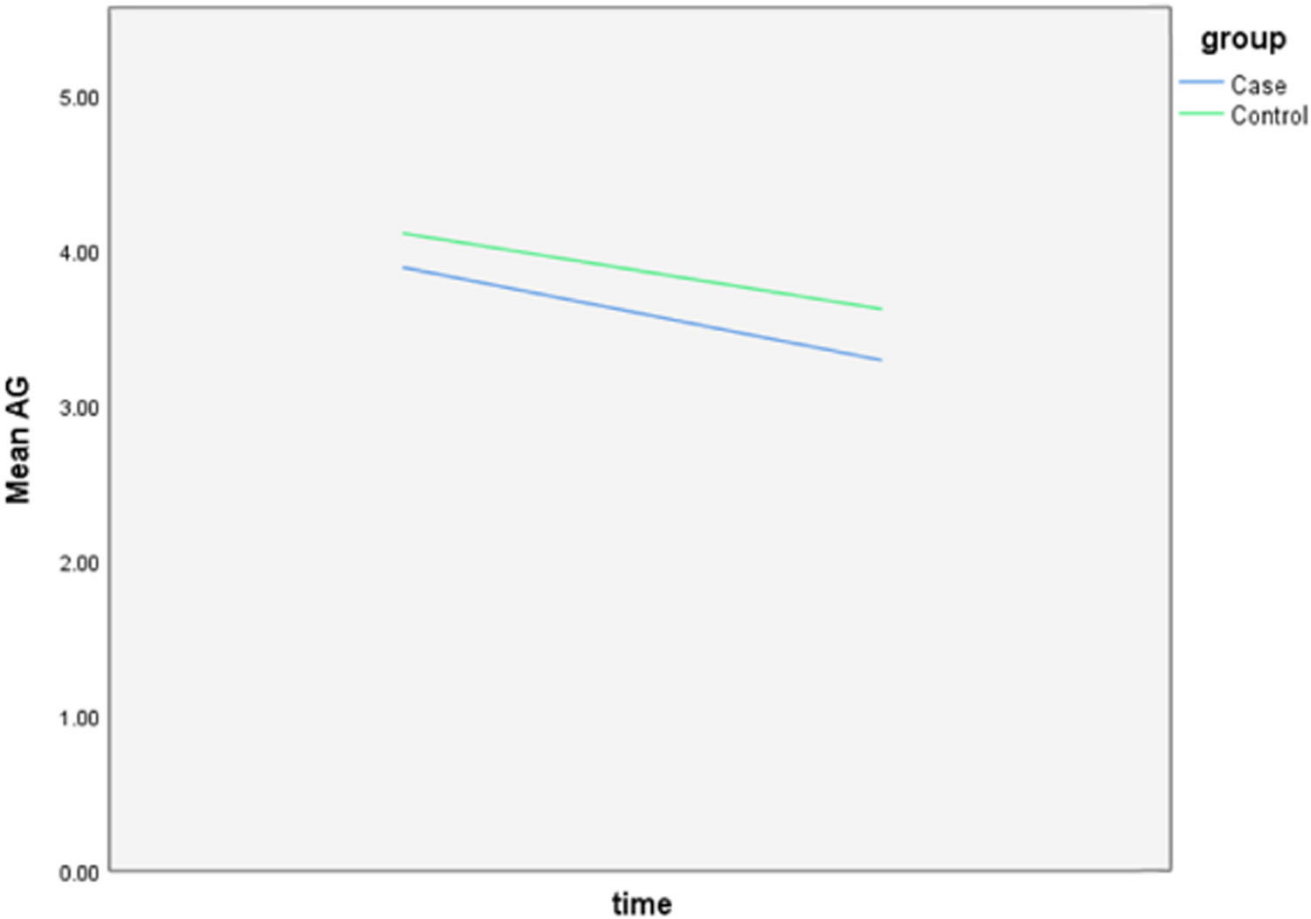
Attached gingiva (AG) alterations throughout the follow‐up period. (0 = Before placement of the mini‐screws, 1, 2, and 3 months following that.)

**Figure 4:**
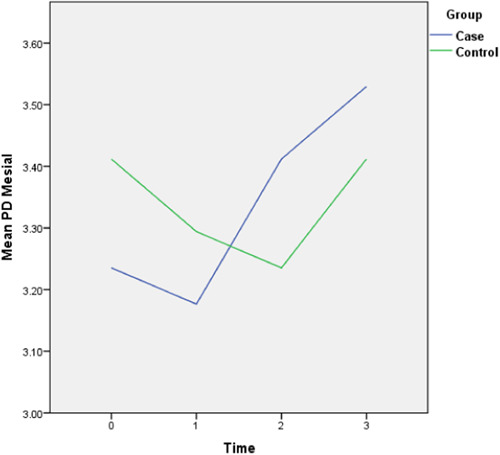
Mesial pocket depth (PD) alterations throughout the follow‐up period. (0 = Before placement of the mini‐screws, 1, 2, and 3 months following that.)

**Figure 5:**
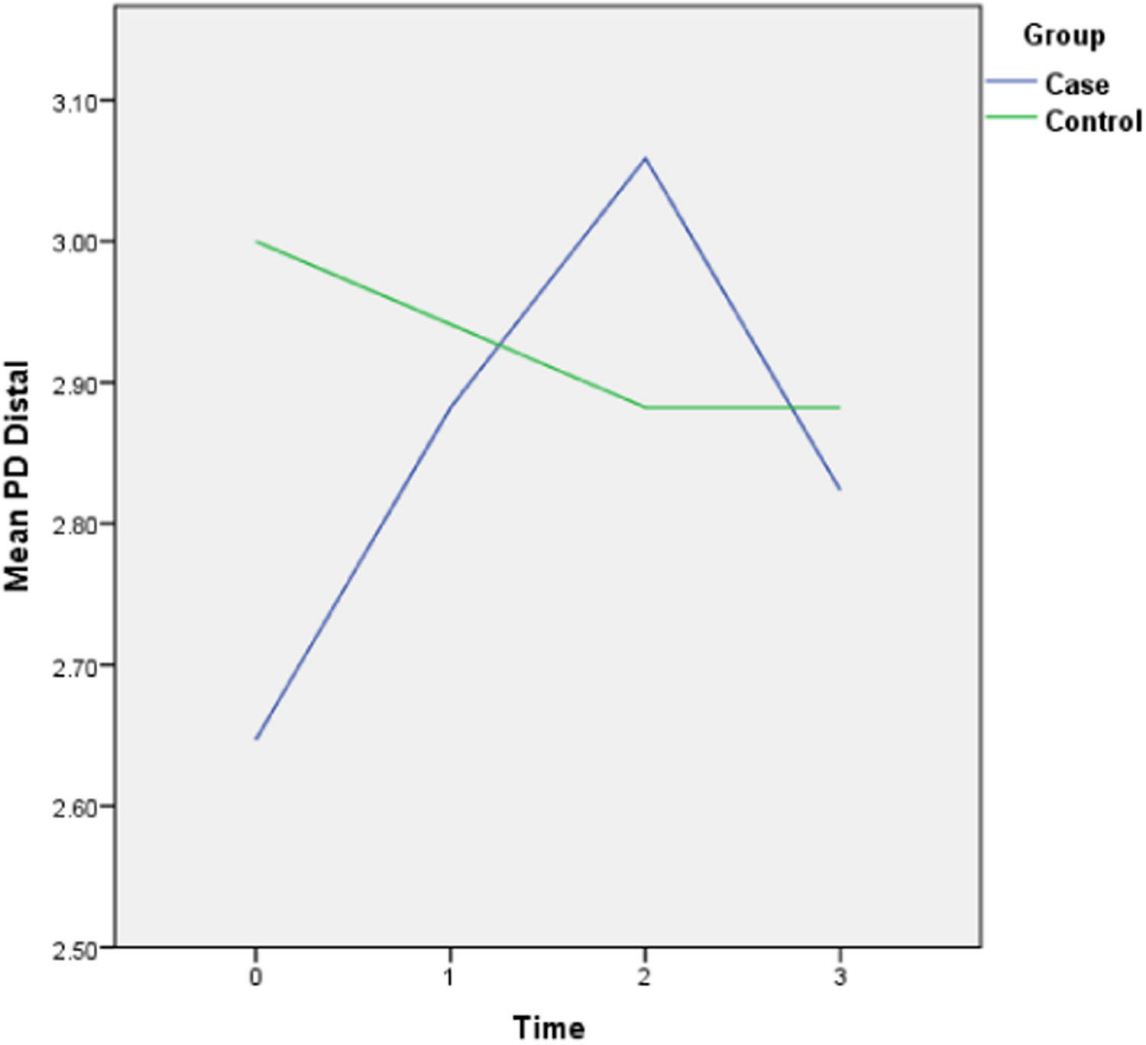
Distal pocket depth (PD) alterations throughout the follow‐up period. (0 = Before placement of the mini‐screws, 1, 2, and 3 months following that.)

**Figure 6:**
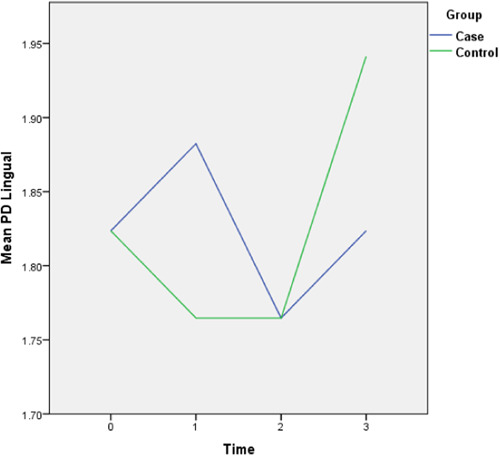
Lingual pocket depth (PD) alterations throughout the follow‐up period. (0 = Before placement of the mini‐screws, 1, 2, and 3 months following that.)

The follow‐up results also revealed that there is only a statistically significant difference between the two groups in the amount of final AG (*p* = 0.028) and the rest of the periodontal variables did not display any significant difference between the two groups. The complete intragroup alterations and intergroup comparisons at different time points are presented in Figures 1, 2, 3, 4, 5, 6.

The PI did not follow any major changes during the 3‐month follow‐up, and following the implantation of the mini‐screws, it increased slightly, which returned to the initial value after 3 months. In the control group, this parameter decreased slightly from 82% to 72%. The GI escalated after the insertion of the mini‐screws by 0.7 units and decreased to the baseline levels during the 2nd and 3rd months and in general did not show any significant changes (Figure 2). This amount for the control group remained steady throughout the follow‐ups. The values of AG were nearly consistent throughout the 3‐month duration (Figure 3). Both baseline and 3‐month follow‐ups revealed significantly higher AG in the control group, nonetheless, there was no significant change from baseline to 3‐month follow‐up within each group. Likewise, there were no remarkable changes in the amount of distal and lingual PD (Figures 5 and 6). Conversely, the mesial PD increased by approximately 0.3 mm compared to the initial amount at the end of 3rd month (Figure 4); however, the changes were not significant.

## 4 DISCUSSION

The present study was conducted aiming to evaluate the periodontal tissue status in the adjacent teeth of the implanted orthodontic mini‐screws to identify the effects of these appliances on the periodontal status of the patients and additionally, to detect possible detrimental impacts on the tissue to enhance the success rate of the orthodontic treatment and in case of any drawbacks, introducing an oral health protocol and follow‐up visits to maintain the periodontal tissue health at the optimum levels. Unfortunately, the number of conducted studies with regard to the effects of mini‐screws on periodontal health is limited and is mostly on animal subjects. Thus, more clinical human studies are required to unravel the mystery of the proposed concern.

Among the monitored variables in this study, a significant difference solely was observed between the two groups in the amount of AG and the other indices did not display any significant alterations. When monitoring the changes during the 3‐month period in both groups, it can be noted that although the difference was significant between the two groups, the amount of AG was constant in both with no significant change from baseline to the last follow‐up. Also, the results of the baseline *t‐*test indicated a significant difference in AG between the two groups. Considering that there were no substantial changes (*p* > 0.05) in this variable during the mentioned period of time within each group, it can be concluded that the AG levels were also constant and stable in both groups.

In similar studies on animals, there is controversial evidence supporting an increase in the amount of AG (Melsen, 2001; Melsen et al., 1988), a decrease (Melsen et al., 1988), and no changes (Murakami et al., 1989). Despite the fact that precise evaluation of the AG requires histological evaluation, due to ethical concerns it cannot be performed in human studies. Thus, similar studies considered the increase in the long junctional epithelium or the formation of connective tissue (Melsen, 2001; Melsen et al., 1988). The escalation in the amount of mesial PD and other regions might be the result of gingival enlargement and not necessarily indicative of a disease.

The results of the present study showed that there were no statistically significant changes throughout the 3‐month follow‐up within each group. Furthermore, despite an increase in the GI and PI, these variables returned to normal levels after 3‐months and even displayed lower values at the end of the visits. These findings are in accordance with those of similar studies (Erverdi et al., 2007; Kuroda et al., 2007; Papageorgiou et al., 2012; Xun et al., 2007); however, it has been indicated that in case of an increase in the amount of GI, it would be a risk factor for the success of the orthodontic treatment (Papageorgiou et al., 2012). Moreover, there is no supporting evidence for the same outcome regarding the PI.

Bayani et al. (2015) reported that not only there would be no negative effects on the periodontal status following the treatment with mini‐screws, but also an improvement in this regard can be anticipated specifically in the amount of AG. On the other hand, Mazin et al. (2016) reported a drastic increase in the amounts of PI, GI, and BOP for the male and female subjects who underwent fixed orthodontics appliances. However, due to the fact that they did not specify the type of fixed appliance in their study, it should be borne in mind that different appliances can display different effects on the adjacent tissue. Thus, validation of the possible drawbacks should be done before the treatment to prevent further damage.

When it comes to the other variables, in the only two available similar studies on human subjects (Bayani et al., 2015; Ghanbari et al., 2015), the authors also supported no significant changes in the BOP, probing pocket depths, keratinized gingiva, and the crestal bone levels; therefore, upholding the findings of our study. Moreover, a finite element analysis on the amount of compressive stress on the PDL of adjacent teeth to the mini‐screws by Albogha and Takahashi (2019) revealed that the placement of mini‐screws within <1 mm space from the root surface would increase the stress to periodontium and PDL and can contribute to alterations in the periodontal health indices.

Overall, it can be suggested that the impact of mini‐screw implants in orthodontic movements would not possibly be a threat to periodontal health if standard oral health maintenance was followed. Moreover, the protocols that are implemented for periodontal health maintenance during the fixed orthodontic treatment, the most important of which is plaque control, seem to be adequate and applicable to treatments using mini‐implants too (Dersot, 2010; Nassar et al., 2013). Mohamed et al. (2023) compared chlorhexidine mouthwash use to a placebo group for 6 months. However, no significant difference was found in the survival rate of mini‐screws nor in the gingival health of subjects. Nevertheless, the scarce nature of the conducted human studies on this topic necessitates further investigation.

The limitation of the present study comprised a limited sample size, the absence of an evaluation of the possible effect of demographic factors of the subject on the results, and other periodontal health indicators such as crestal bone levels. Therefore, future studies on this topic addressing the mentioned limitations would be beneficial.

## 5 CONCLUSIONS

Within its limitations, the present study indicates that the periodontal health status of the adjacent teeth to the implanted mini‐screws for orthodontic anchorage does not alter significantly during the 3‐month follow‐up, compared to the teeth in the control group. Thus, the orthodontic mini‐screws can be utilized as a safe anchorage unit when required, on condition that the oral hygiene levels are kept at the standard levels.

## AUTHOR CONTRIBUTIONS

**Negar Moeini**: Conceptualization; data collection; manuscript writing. **Hamoun Sabri**: Manuscript writing; statistical analysis; data interpretation. **Pablo Galindo‐Fernandez**: Visualization; software. **Hoorieh Mirmohamadsadeghi**: Conceptualization; critical review and final approval. **Nasrin Keshavarz Valian**: Conceptualization; critical review and final approval.

## CONFLICT OF INTEREST STATEMENT

The authors declare no conflict of interest.

## ETHICS STATEMENT

Shahid Beheshti University of Medical Sciences Research Institute: IR. SBMU. DRC. REC.1398.137

## Supporting information

Supporting information.

## Data Availability

The data of this research study would be provided upon reasonable request from the corresponding author.

## References

[cre2757-bib-0001] Abdulnabi, Y. , Albogha, M. H. , Abuhamed, H. , & Kaddah, A. (2017). Non‐surgical treatment of anterior open bite using miniscrew implants with posterior bite plate. Orthodontic Waves, 76, 40–45.

[cre2757-bib-0002] Albogha, M. H. , & Takahashi, I. (2019). Effect of loaded orthodontic miniscrew implant on compressive stresses in adjacent periodontal ligament. The Angle Orthodontist, 89, 235–241. 30230377 10.2319/122017-873.1 PMC8120879

[cre2757-bib-0003] Alfuriji, S. , Alhazmi, N. , Alhamlan, N. , Al‐Ehaideb, A. , Alruwaithi, M. , Alkatheeri, N. , & Geevarghese, A. (2014). The effect of orthodontic therapy on periodontal health: A review of the literature. International Journal of Dentistry, 2014, 1–8. 10.1155/2014/585048 PMC4060421 24991214

[cre2757-bib-0004] Alharbi, F. , Almuzian, M. , & Bearn, D. (2018). Miniscrews failure rate in orthodontics: Systematic review and meta‐analysis. European Journal of Orthodontics, 40, 519–530. 29315365 10.1093/ejo/cjx093

[cre2757-bib-0005] Atassi, F. , & Awartani, F. (2010). Oral hygiene status among orthodontic patients. The Journal of Contemporary Dental Practice, 11, 025–032. 20953561

[cre2757-bib-0006] Bae, S. M. , Park, H. S. , Kyung, H. M. , Kwon, O. W. , & Sung, J. H. (2002). Clinical application of micro‐implant anchorage. Journal of Clinical Orthodontics: JCO, 36, 298–302. 12056211

[cre2757-bib-0007] Baek, S. H. , Kim, B. M. , Kyung, S. H. , Lim, J. K. , & Kim, Y. H. (2008). Success rate and risk factors associated with mini‐implants reinstalled in the maxilla. The Angle Orthodontist, 78, 895–901. 18298220 10.2319/091207-430.1

[cre2757-bib-0008] Bayani, S. , Heravi, F. , Radvar, M. , Anbiaee, N. , & Madani, A. (2015). Periodontal changes following molar intrusion with miniscrews. Dental Research Journal, 12, 379–385. 26288629 10.4103/1735-3327.161462 PMC4533198

[cre2757-bib-0009] Casaña‐Ruiz, M. D. , Bellot‐Arcís, C. , Paredes‐Gallardo, V. , García‐Sanz, V. , Almerich‐Silla, J. M. , & Montiel‐Company, J. M. (2020). Risk factors for orthodontic mini‐implants in skeletal anchorage biological stability: A systematic literature review and meta‐analysis. Scientific Reports, 10, 5848. 32246125 10.1038/s41598-020-62838-7 PMC7125198

[cre2757-bib-0010] Chapple, I. L. C. , Mealey, B. L. , Van Dyke, T. E. , Bartold, P. M. , Dommisch, H. , Eickholz, P. , Geisinger, M. L. , Genco, R. J. , Glogauer, M. , Goldstein, M. , Griffin, T. J. , Holmstrup, P. , Johnson, G. K. , Kapila, Y. , Lang, N. P. , Meyle, J. , Murakami, S. , Plemons, J. , Romito, G. A. , … Yoshie, H. (2018). Periodontal health and gingival diseases and conditions on an intact and a reduced periodontium: Consensus report of workgroup 1 of the 2017 world workshop on the classification of periodontal and peri‐implant diseases and conditions. Journal of Periodontology, 89, S74–S84. 29926944 10.1002/JPER.17-0719

[cre2757-bib-0011] Chen, Y. H. , Chang, H. H. , Chen, Y. J. , Lee, D. , Chiang, H. H. , & Yao, C. C. (2008). Root contact during insertion of miniscrews for orthodontic anchorage increases the failure rate: An animal study. Clinical Oral Implants Research, 19, 99–106. 17956568 10.1111/j.1600-0501.2007.01418.x

[cre2757-bib-0012] Cousley, R. R. J. , & Sandler, P. J. (2015). Advances in orthodontic anchorage with the use of mini‐implant techniques. British Dental Journal, 218, E4. 25686460 10.1038/sj.bdj.2015.53

[cre2757-bib-0013] Dersot, J. M. (2010). Le contrôle de plaque, un élément essentiel du succès du traitement orthodontique. L'Orthodontie Française, 81, 33–39. 10.1051/orthodfr/2010001 20359447

[cre2757-bib-0014] Erverdi, N. , Usumez, S. , Solak, A. , & Koldas, T. (2007). Noncompliance open‐bite treatment with zygomatic anchorage. The Angle Orthodontist, 77, 986–990. 18004921 10.2319/101206-422.1

[cre2757-bib-0015] Ghanbari, H. O. , Gharechahi, M. , Ghanbarzadeh, M. , Nikkhaah Raankoohi, A. , & Dastmalchi, P. (2015). Evaluation of periodontal condition in intruded molars using miniscrews. Journal of Dental Materials and Techniques, 4, 145–152.

[cre2757-bib-0017] Imano, M. , Spada, P. P. , Macalossi, J. M. S. , & Deliberador, T. M. (2020). Treatment of localized gingival recession by means tunnel technique after the orthodontic treatment. A follow‐up of 1 year. Case Reports in Dentistry, 2020, 1–6. 10.1155/2020/8816510 PMC7495163 32963839

[cre2757-bib-0018] Karkhanechi, M. , Chow, D. , Sipkin, J. , Sherman, D. , Boylan, R. J. , Norman, R. G. , Craig, R. G. , & Cisneros, G. J. (2013). Periodontal status of adult patients treated with fixed buccal appliances and removable aligners over one year of active orthodontic therapy. The Angle Orthodontist, 83, 146–151. 22725616 10.2319/031212-217.1 PMC8805524

[cre2757-bib-0019] Kravitz, N. D. , & Kusnoto, B. (2007). Risks and complications of orthodontic miniscrews. American Journal of Orthodontics and Dentofacial Orthopedics: Official Publication of the American Association of Orthodontists, Its Constituent Societies, and the American Board of Orthodontics, 131, 43–51. 10.1016/j.ajodo.2006.04.027 17448385

[cre2757-bib-0020] Kuroda, S. , Sugawara, Y. , Tamamura, N. , & Takano‐Yamamoto, T. (2007). Anterior open bite with temporomandibular disorder treated with titanium screw anchorage: Evaluation of morphological and functional improvement. American Journal of Orthodontics and Dentofacial Orthopedics, 131, 550–560. 17418724 10.1016/j.ajodo.2006.12.001

[cre2757-bib-0021] Leung, M. T. , Lee, T. C. , Rabie, A. B. , & Wong, R. W. (2008). Use of miniscrews and miniplates in orthodontics. Journal of Oral and Maxillofacial Surgery: Official Journal of the American Association of Oral and Maxillofacial Surgeons, 66, 1461–1466. 18571031 10.1016/j.joms.2007.12.029

[cre2757-bib-0046] Liu, J.‐N. , He, Y.‐X. , Jia, X.‐T. , Huang, R. , Zeng, N. , Fan, X.‐C. , & Huang, X.‐F. (2023). Feasibility of mini‐implant insertion between mesial and distal buccal roots of a maxillary first molar: A cone‐beam computed tomography imaging study. American Journal of Orthodontics and Dentofacial Orthopedics . Advance online publication. 10.1016/j.ajodo.2023.03.024 37318427

[cre2757-bib-0022] Löe, H. , & Silness, J. (1963). Periodontal disease in pregnancy I. Prevalence and severity. Acta Odontologica Scandinavica, 21, 533–551. 14121956 10.3109/00016356309011240

[cre2757-bib-0023] Mazin, H. , Ali, S. S. , & Salah, R. (2016). The Effect of Fixed Orthodontic Appliances on Gingival Health.

[cre2757-bib-0024] Melsen, B. (2001). Tissue reaction to orthodontic tooth movement—a new paradigm. The European Journal of Orthodontics, 23, 671–681. 11890063 10.1093/ejo/23.6.671

[cre2757-bib-0025] Melsen, B. , Agerbæk, N. , Erikson, J. , & Terp, S. (1988). New attachment through periodontal treatment and orthodontic intrusion. American Journal of Orthodontics and Dentofacial Orthopedics, 94, 104–116. 3165238 10.1016/0889-5406(88)90358-7

[cre2757-bib-0026] Miethke, R.‐R. , & Vogt, S. (2005). A comparison of the periodontal health of patients during treatment with the Invisalign® system and with fixed orthodontic appliances. Journal of Orofacial Orthopedics/Fortschritte der Kieferorthopädie, 66, 219–229. 15959635 10.1007/s00056-005-0436-1

[cre2757-bib-0027] Mohamed, A. , Wafaie, K. , Mohammed, H. , Mohamed, A. M. A. , Xinrui, W. , Vandevska‐Radunovic, V. , & Yiqiang, Q. (2023). Effect of chlorhexidine mouthwash on gingival health around orthodontic miniscrew implants: A pilot placebo‐controlled randomized trial. Orthodontics & ‐Craniofacial Research, 26, 163–170. 35751508 10.1111/ocr.12596

[cre2757-bib-0028] Molina‐Solana, R. , Yáñez‐Vico, R. M. , Iglesias‐Linares, A. , Torres‐Lagares, D. , & Solano‐Reina, E. (2013). Miniscrew appliances and their use in orthodontics. Open Journal of Stomatology, 3, 6.

[cre2757-bib-0029] Moon, C.‐H. , Lee, D.‐G. , Lee, H.‐S. , Im, J.‐S. , & Baek, S. H. (2008). Factors associated with the success rate of orthodontic miniscrews placed in the upper and lower posterior buccal region. The Angle Orthodontist, 78, 101–106. 18193973 10.2319/121706-515.1

[cre2757-bib-0030] Murakami, T. , Yokota, S. , & Takahama, Y. (1989). Periodontal changes after experimentally induced intrusion of the upper incisors in *Macaca fuscata* monkeys. American Journal of Orthodontics and Dentofacial Orthopedics, 95, 115–126. 2916468 10.1016/0889-5406(89)90390-9

[cre2757-bib-0031] Nassar, P. O. , Bombardelli, C. G. , Walker, C. S. , Neves, K. V. , Tonet, K. , Nishi, R. N. , Bombonatti, R. , & Nassar, C. A. (2013). Periodontal evaluation of different toothbrushing techniques in patients with fixed orthodontic appliances. Dental Press Journal of Orthodontics, 18, 76–80. 10.1590/s2176-94512013000100017 23876953

[cre2757-bib-0032] Ong, M. M. A. , & Wang, H.‐L. (2002). Periodontic and orthodontic treatment in adults. American Journal of Orthodontics and Dentofacial Orthopedics, 122, 420–428. 12411890 10.1067/mod.2002.126597

[cre2757-bib-0033] Papageorgiou, S. N. , Zogakis, I. P. , & Papadopoulos, M. A. (2012). Failure rates and associated risk factors of orthodontic miniscrew implants: A meta‐analysis. American Journal of Orthodontics and Dentofacial Orthopedics, 142, 577‐595. 23116500 10.1016/j.ajodo.2012.05.016

[cre2757-bib-0034] Prabhu, J. , & Cousley, R. R. (2006). Current products and practice: Bone anchorage devices in orthodontics. Journal of Orthodontics, 33, 288–307. 17142335 10.1179/146531205225021807

[cre2757-bib-0035] Rodriguez, J. C. , Suarez, F. , Chan, H.‐L. , Padial‐Molina, M. , & Wang, H.‐L. (2014). Implants for orthodontic anchorage: Success rates and reasons of failures. Implant Dentistry, 23, 155–161. 24614877 10.1097/ID.0000000000000048

[cre2757-bib-0036] Sadowsky, C. , & BeGole, E. A. (1981). Long‐term effects of orthodontic treatment on periodontal health. American Journal of Orthodontics, 80, 156–172. 6943936 10.1016/0002-9416(81)90216-5

[cre2757-bib-0037] Severo, F. C. , & Barbosa, G. F. (2015). Risk factors and success rates associated with orthodontic mini‐implants: A literature review. Revista Odonto Ciencia, 30, 200–204.

[cre2757-bib-0038] Viwattanatipa, N. , Thanakitcharu, S. , Uttraravichien, A. , & Pitiphat, W. (2009). Survival analyses of surgical miniscrews as orthodontic anchorage. American Journal of Orthodontics and Dentofacial Orthopedics, 136, 29–36. 19577145 10.1016/j.ajodo.2007.06.018

[cre2757-bib-0039] Xun, C. , Zeng, X. , & Wang, X. (2007). Microscrew anchorage in skeletal anterior open‐bite treatment. The Angle Orthodontist, 77, 47–56. 17029531 10.2319/010906-14R.1

[cre2757-bib-0040] Yanagita, T. , Kuroda, S. , Takano‐Yamamoto, T. , & Yamashiro, T. (2011). Class III malocclusion with complex problems of lateral open bite and severe crowding successfully treated with miniscrew anchorage and lingual orthodontic brackets. American Journal of Orthodontics and Dentofacial Orthopedics: Official Publication of the American Association of Orthodontists, Its Constituent Societies, and the American Board of Orthodontics, 139, 679–689. 21536212 10.1016/j.ajodo.2009.07.023

